# Identifying differential exon splicing using linear models and correlation coefficients

**DOI:** 10.1186/1471-2105-10-26

**Published:** 2009-01-20

**Authors:** Sonia H Shah, Jacqueline A Pallas

**Affiliations:** 1 Bloomsbury Centre for Bioinformatics, Department of Computer Science, University College London, Gower Street, London, UK; 2 Wolfson Institute of Biomedical Research, University College London, Gower Street, London, UK

## Abstract

**Background:**

With the availability of the Affymetrix exon arrays a number of tools have been developed to enable the analysis. These however can be expensive or have several pre-installation requirements. This led us to develop an analysis workflow for analysing differential splicing using freely available software packages that are already being widely used for gene expression analysis. The workflow uses the packages in the standard installation of R and Bioconductor (BiocLite) to identify differential splicing. We use the splice index method with the LIMMA framework. The main drawback with this approach is that it relies on accurate estimates of gene expression from the probe-level data. Methods such as RMA and PLIER may misestimate when a large proportion of exons are spliced. We therefore present the novel concept of a gene correlation coefficient calculated using only the probeset expression pattern within a gene. We show that genes with lower correlation coefficients are likely to be differentially spliced.

**Results:**

The LIMMA approach was used to identify several tissue-specific transcripts and splicing events that are supported by previous experimental studies. Filtering the data is necessary, particularly removing exons and genes that are not expressed in all samples and cross-hybridising probesets, in order to reduce the false positive rate. The LIMMA approach ranked genes containing single or few differentially spliced exons much higher than genes containing several differentially spliced exons. On the other hand we found the gene correlation coefficient approach better for identifying genes with a large number of differentially spliced exons.

**Conclusion:**

We show that LIMMA can be used to identify differential exon splicing from Affymetrix exon array data. Though further work would be necessary to develop the use of correlation coefficients into a complete analysis approach, the preliminary results demonstrate their usefulness for identifying differentially spliced genes. The two approaches work complementary as they can potentially identify different subsets of genes (single/few spliced exons vs. large transcript structure differences).

## Background

The Affymetrix Exon 1.0ST arrays contain approximately 5.5 million probes which are grouped into 1.4 million probesets, targeting over 1 million exons. The data generated from these probe signals can be summarised into probeset signals to provide a measure of expression of individual exons. These probesets in turn can be assembled into virtual transcript clusters based on annotations of gene structure. By combining signals from probesets mapping to the same transcript cluster, an expression measure for that transcript cluster (gene) can be calculated. The arrays make it possible to observe differential exon inclusion or skipping, therefore providing an extra dimension of genomic information beyond the classical gene expression results from microarrays [1].

### Gene-level summary and analysis

The main challenge for gene-level analysis is estimating reliable gene expression measures from the probe signals. Probesets are associated only with exons and the grouping of exon probesets into genes is a dynamic post-design process [2] based on genome annotations obtained from sources of varying quality (RefSeq genes, mRNAs and ESTS from Genbank, and predictions by *ab-initio *methods such as GENSCAN). The array design includes probesets targeting predicted exons, which though have lower confidence annotations, enable the detection of novel exons and splicing events. However, including such probesets to estimate gene expression can have a negative as exons with low confidence annotation, such as computational exon predictions, will have a lower probability of being present in the cell compared with well-annotated exons. Affymetrix have therefore classed probesets into 3 categories: core probesets (based on RefSeq transcripts and full-length mRNAs), extended probesets (this includes the core probesets plus probesets mapping to exons with cDNA-based annotations) and full probesets (includes the core and extended probesets as well as those mapping to exons with *ab initio *predictions). Analysis of exon array data by Xing *et al*. [3] suggests that extended and full probes are usually poor indicators of overall gene expression. Estimating gene-level expression is further affected by the degree of alternative splicing and the number of cross-hybridising probes (probes that hybridise to sequences other than the target sequence). If a gene contains a large number of core probesets that target alternatively spliced regions this may lead to under-estimation of overall gene expression, while a large number of cross-hybridising probesets may lead to over-estimation of overall gene expression.

There are two commonly used algorithms for generating gene-level summaries from the raw exon array data: RMA (Robust Multichip Average) [4] and PLIER (Probe Logarithmic Intensity Error) [5]. These are implemented in the Affymetrix Power Tools (APT) software which is freely available from the Affymetrix website. APT allows the selection of either the core, extended or full Affymetrix transcript cluster annotations for gene-level signal estimation. The Affymetrix transcript cluster library files, which are used by APT to determine which probesets should be used for generating gene signals, only include unique probesets. This reduces errant gene signal estimations due to cross-hybridising probes. Robust methods such as RMA and PLIER should be minimally affected by a limited amount of alternative splicing at a particular locus [6], but are still prone to misestimates when a large number of exons are differentially spliced.

Once the gene-level summaries have been generated, the data can be analysed to identify differentially expressed genes between sample groups, using a statistical method of choice. A very popular Bioconductor [7] package used for differential expression analysis is the Linear Models for Microarray Analysis (LIMMA) [8]. The package simplifies the analysis of complex experiments by using linear models to analyse the data, making it possible to simultaneously carry out multiple comparisons between sample groups. The analysis uses a moderated t-statistic which has the same interpretation as an ordinary t-statistic except that the standard errors are adjusted using an empirical Bayes method, making the analyses stable even for experiments with small array numbers [9]. A differential gene analysis using LIMMA on RMA-summarised tissue data from the Human Genome U133 Plus 2.0, Human Exon 1.0ST and Human Gene 1.0ST arrays showed that gene-level reproducibility and differential expression detection are quite similar across the three platforms [10].

### Alternative splicing analysis

The simplest method to detect alternative splicing is the Splice Index (SI) method [11]. This assumes that in the absence of splicing the observed signal from each exon will have a constant ratio with the observed signal from the corresponding gene. The exon/gene (probeset/transcript cluster) ratios are referred to as gene-normalised exon intensities. If this ratio differs between two groups it is indicative of splicing. Several tools are available, including APT, which apply an ANOVA model to the gene-normalised exon intensities. In the case of two sample groups the ANOVA model reduces to a t-test. For multiple sample groups, the ANOVA model will provide only a single p-value indicating the probability that a transcript has differential alternative splicing. To determine in which sample groups the exon splicing is occurring either a post-hoc Tukey analysis can be done or a contrast matrix including all comparisons can be constructed. The LIMMA Package in Bioconductor has the framework for the latter, and conveniently reports statistics for all comparisons as well as handling the multiple testing problem. LIMMA also uses an emperical Bayes approach for estimating sample variances. The moderated t-statistic calculated by LIMMA is more robust than the ordinary t-statistic with small sample sizes [8]. For these reasons we chose to use LIMMA instead of ANOVA. It is already a widely used package but so far has only been used for differential gene expression analysis. In this paper we propose a workflow for detecting differential exon splicing using LIMMA. The workflow is designed to reduce false positives by including filtering steps, which are discussed later. APT is used initially to generate the gene and exon summaries, as the software has been optimised for this memory-intensive step. All subsequent steps in the workflow are carried out using R and Bioconductor. The packages included in the standard Bioconductor (BiocLite) installation are sufficient to carry out the alternative splicing analysis and no additional packages, software or database installations are required.

The use of the SI method with LIMMA (this will be referred to as SI/LIMMA), though successful at identifying tissue-specific splicing events, was prone to false positives resulting from inaccurate gene-level estimations. The SI/LIMMA method is also sensitive to large differences in gene expression level between sample groups which can amplify the noise [12]. To tackle this issue we introduce the concept of a gene correlation coefficient to identify differential gene splicing. This approach was proposed after observing that for many genes the probesets expression signals were found to be highly variable (likely due to a combination of exon splicing, cross-hybridising probes, non-responsive probes and presence of SNPs within the targeting sequence), but the expression pattern of the probesets across a gene was highly correlated in all samples when differential splicing was absent i.e. if a probeset had a lower intensity signal than the adjacent 5' probeset but higher than the adjacent 3' probeset in one sample, this pattern was seen in all other samples. In the presence of differential splicing, the probeset expression pattern for a gene will be disrupted in one group leading to a decrease in correlation between the two groups. We hypothesise that in the absence of differential splicing we would expect a correlation coefficient close to 1. Differences in splicing and therefore differences in probeset signal pattern between the two groups will result in lower correlation. The advantage of using a correlation coefficient approach is that it does not require gene-level estimates.

We applied the SI/LIMMA and correlation methods to the Affymetrix human tissue public dataset (available from ) to identify tissue-specific splice events. The data set consists of 33 samples (11 tissue samples, 3 technical replicates per tissue). The LIMMA analysis identified several tissue-specific splicing events that were supported by previous experimental studies. These tissue-specific splice events were also found to have the lowest correlation coefficients in the respective tissue comparisons. We also compare results from previous exon array studies. Our results indicate that the SI/LIMMA approach is better for identifying genes that have only single or few exons differentially spliced while the correlation approach is better for identifying larger differences in transcript structures. The SI/LIMMA method also works better when probeset expression within a gene has low variability as this allows for more robust estimations of gene signals while the correlation coefficient obviously works well when probeset expression within a gene is highly variable. The two methods can therefore work complimentary to identify different subsets of differentially spliced genes.

## Results

### Using LIMMA to Identify Splice Events

**Figure 1:**
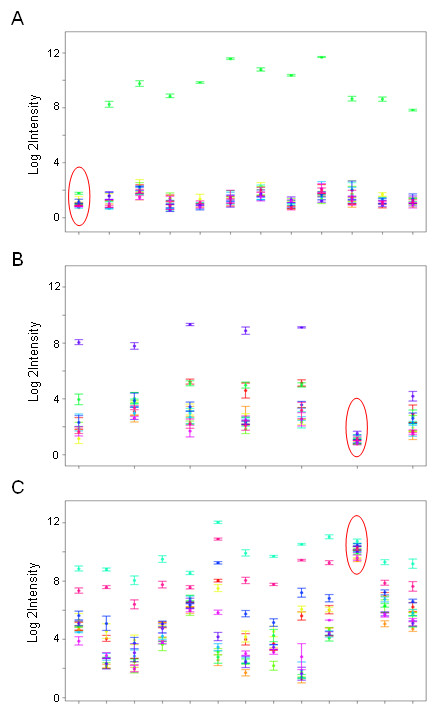
**False Positives**. Examples of probesets falsely identified as differentially spliced. The expression plot shows the mean log 2 intensities of the core probesets in each tissue with standard error bars. The probesets are sorted by genomic location from 5' to 3'. Circled probesets had Benjamini-Hochberg-corrected p-values less than 0.0001. A) and B) False positive due to non-responsive or unexpressed exon in all samples and C) cross-hybridising probeset.

Gene and exon-level summaries were generated using the RMA algorithm implemented in APT. We restricted our analysis to identify differential splicing of well-annotated exons and therefore used only core probesets and transcript clusters for generating gene- and exon-level summaries. To find statistically significant tissue-specific splicing events, LIMMA was applied to the gene-normalised exon intensities, which were calculated in R. Eleven contrasts were made simultaneously (e.g. breast vs. non-breast, heart vs. non-heart etc.). For each probeset in each contrast LIMMA reported the log fold change (where a positive value indicates exon retention and a negative value indicates exon splicing in one group relative to the other), moderated t-statistic, the raw p-values and the adjusted p-values (to account for multiple testing). Probesets with Benjamini-Hochberg-adjusted p-values less than 0.0001 were considered statistically significant. 34208 probesets mapping to 10122 transcript clusters passed the stringent p-value threshold in at least one out of the eleven comparisons. Upon visual inspection of the exon expression levels using expression plots, a large number of the significant probesets were found to be false positives. These probesets were misidentified (a) when a probeset is non-responsive or an exon is not expressed in all samples (spliced out in all sample) (Figure 1A and 1B) and when a probeset is cross-hybridising (Figure 1C).

### Filtering expression data to reduce false positives

**Figure 2:**
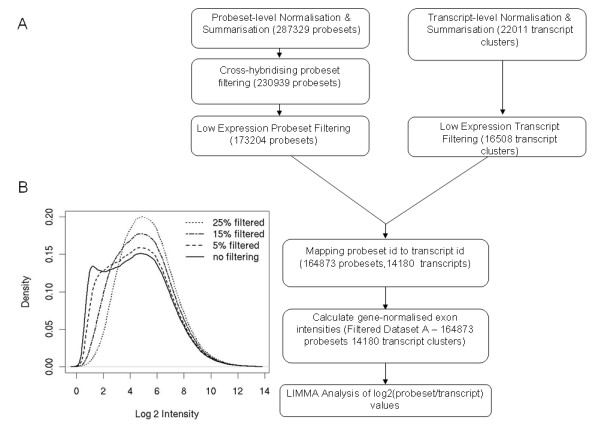
**Analysis Workflow**. A) LIMMA analysis workflow where the probeset and transcript-level summarisation and normalisation is carried out using the Affymetrix Power Tools. All subsequent steps are carried out using R and Bioconductor. B) The effect of filtering low-expressed probesets on the intensity distribution in one of the breast samples. When 25% of the probesets found in the lower quartile in all samples are filtered out, the intensity distribution has a more normal distribution.

To reduce false predictions of alternative splicing events filtering steps were applied to the data prior to LIMMA analysis:

1. Affymetrix has categorised probesets as either unique (perfectly match only the target sequence), similar (perfectly match more than one sequence) or mixed (perfectly or partially match more than one sequence). Probesets that are not unique were removed from the exon-level data.

2. To reduce false positives caused by unexpressed genes and/or exons spliced out in all samples, a simple approach was used where probesets and transcript clusters that were found in the lower quartile of the intensity distribution in all 33 samples were filtered out (Figure 2B).

3. Despite the fact that the Splicing Index approach is designed to mitigate differences in gene expression level, previous experience has shown that very large differences in gene expression level can amplify the noise leading to potential false positives [12]. Therefore, the analysis was also done on a subset of low variance genes. Transcript clusters found in the quartile containing the highest sample-to-sample variation were excluded.

The workflow incorporating these steps is shown in Figure 2. The LIMMA analysis was performed on unfiltered data, data filtered for cross-hybridising probesets and low/unexpressed probesets and transcript clusters (this will be referred to as **filtered dataset A**) and data using a subset of low-variant transcript clusters from the filtered dataset A (this will be referred to as **filtered dataset B**).

The number of probesets with Benjamini-Hochberg-adjusted p-values less than 0.0001, for each data set, is shown in Table 1. More than half (57%) of the probesets that had passed the p-value cut-off in the unfiltered data were removed as they were either cross-hybridising or had very low signal in all samples (likely to be unexpressed genes and noise). We used the Affymetrix annotation files to obtain probeset and gene annotation. Expression plots and transcript structures from the X:MAP genome browser [13] (based on the Ensembl database) were used to map probesets to exons and to try and deduce which transcripts were being expressed in the different tissues.

**Table 1:** Number of significant probesets

	Unfiltered data	Filtered dataset A	Filtered dataset B
Breast-specific	1725	669	273

Cerebellum-specific	12009	5890	3122

Heart-specific	2746	1492	476

Kidney-specific	3354	1382	377

Liver-specific	4956	2213	636

Muscle-specific	3153	1606	583

Pancreas-specific	4908	1984	817

Prostate-specific	2494	1292	531

Spleen-specific	1513	674	332

Testes-specific	10430	2819	1476

Thyroid-specific	897	387	112

Total no. of significant probesets	34208	14536	6895

Total no. of transcript clusters analysed	17881	14180	10414

Total no. of transcript clusters with at least one significant probeset	10122	6338	3564

The table shows the number of probesets with Benjamini-Hochberg-corrected p-values less than 0.0001 in each of the 11 comparisons for the unfiltered and filtered datasets. The table also shows the number of transcript clusters (genes) that were found to contain at least one significant probeset.

Several known tissue-specific splice events were identified in all 3 datasets, with their significance ranking and p-values greatly improving when the data was filtered (Table 2). To validate some of the splicing events identified by the analysis, we tried to map the expression data to known transcript structures and searched for literature evidence supporting the tissue-specific expression of the exons. Below are examples of 5 genes identified by SI/LIMMA to have tissue-specific splicing in at least one tissue

**Table 2:** Significant Splicing Events

Affymetrix Probeset ID	Gene and exon	LIMMA Comparison	Rank in analysis of unfiltered dataset (Benjamini-Hochberg-corrected p-value)	Rank with analysis of filtered dataset A (Benjamini-Hochberg-corrected p-value)	Rank with analysis of filtered dataset B (Benjamini-Hochberg-corrected p-value)
3400083	WNK1 exon 3 ENSE00000437674	Kidney vs. non-kidney	53 (1.7e-12)	11 (1.9e-12)	1 (7.0e-12)

3400090	WNK1 exon 4 ENSE00000711974	Kidney vs. non-kidney	82 (1.1e-11)	21 (1.5e-11)	4 (5.5e-11)

3400056	WNK1 exon 1 ENSE000001527902	Kidney vs. non-kidney	161 (1.3e-10)	44 (1.7e-10)	9 (5.5e-10)

3400080	WNK1 exon 2 ENSE00000711927	Kidney vs. non-kidney	507 (2.2e-08)	169 (3.3e-08)	35 (1.1e-07)

3427830	SLC25A3 exon3B ENSE00000753648	Muscle vs. non-muscle	25 (5.9e-13)	3 (1.3e-12)	2 (1.2e-12)

3427830	SLC25A3 exon3B ENSE00000753648	Heart vs. non-heart	106 (1.7e-11)	41 (1.1e-11)	8 (3.6e-11)

3427827	SLC25A3 exon 3A ENSE00000753647	Heart vs. non-heart	236 (5.2e-10)	112 (5.8e-10)	26 (1,8e-09)

3427827	SLC25A3 exon 3A ENSE00000753647	Muscle vs. non-muscle	263 (1.6e-09)	126 (1.7e-09)	35 (4.6e-09)

3427827	SLC25A3 exon 3A ENSE00000753647	Thyroid vs. non-thyroid	308 (3.9e-07)	114 (1.2e-09)	22 (2.2e-06)

3918911	ITSN exon 40 ENSE00001488185	Cereb. vs. non-cereb	480 (1.6e-12)	225 (1.7e-12)	63 (4.4e-12)

3918909	ITSN exon 40 ENSE00001488185	Cereb. vs. non-cereb	1822 (1.7e-09)	873 (2.0e-09)	353 (3.6e-09)

3918908	ITSN exon 40 ENSE00001488185	Cereb. vs. non-cereb	1884 (2.1e-09)	885 (2.1e-09)	359 (3.8e-09)

3918903	ITSN exon 35 ENSE00001488224	Cereb. vs. non-cereb	2304 (6.6e-09)	1107 (7.2e-09)	466 (1.2e-08)

2562711	IMMT exon 6 ENSE00000768006	Heart vs. non-heart	37 (4.5e-13)	17 (3.7e-13)	1 (4.3e-12)

2319719	KIF1B exon 20 ENSE00001472763	Heart vs. non-heart	129 (3.6e-11)	55 (3.1e-11)	Not present in dataset B

2319721	KIF1B exon 20 ENSE00001472763	Heart vs. non-heart	134 (4.4e-11)	61 (4.2e-11)	Not present in dataset B

2319722	KIF1B exon 20 ENSE00001472763	Cereb. vs. non-cereb	486 (1.8e-12)	218 (1.7e-12)	Not present in dataset B

2319721	KIF1B exon 20 ENSE00001472763	Cereb. vs. non-cereb	1784 (1.4e-09)	830 (1.4e-09)	Not present in dataset B

Table showing the effect of filtering on the ranking of real splice events. All the probesets in the table had a Benjamini-Hochberg-corrected p-value less than 0.0001 and for each there is literature evidence of the identified splicing events.

#### WNK lysine deficient protein kinase 1 (*WNK-1*)

*WNK-1 *codes for a serine-threonine protein kinase which controls sodium and chloride ion transport and it is known to be expressed in a wide variety of tissues [14]. The kidney vs. non-kidney LIMMA analysis of filtered dataset B identified 7 probesets with Benjamini-Hochberg-corrected p-values less than 0.0001, all of which mapped to exons 1–4 of the *WNK-1 *gene (Table 2). Three of these ranked within the top 10 most significant probesets in the kidney vs. non-kidney comparison of the filtered dataset B. The expression plot for this gene (Additional file 1) suggests that the dominant transcript in kidney lacks the first four exons. In human and mouse, multiple WNK1 mRNA species are expressed that arise by alternative promoter usage and splicing [15,16]. In the kidney, in addition to the originally described "long" WNK1 (L-WNK1) [15], the most prominently expressed WNK1 is a shorter transcript [15,16]. This shorter kidney-specific WNK1 (KS-WNK1) is initiated at an alternative promoter and lacks the first four exons that include the kinase domain of WNK1 [15].

#### Solute carrier family 25, member 3 (*SLC25A3*)

**Figure 3:**
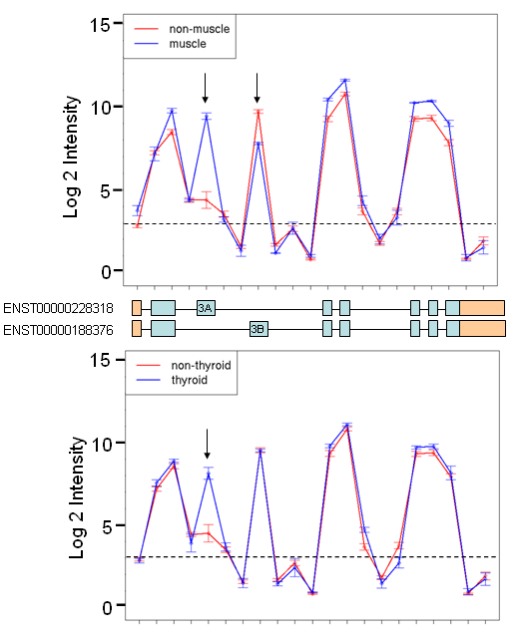
**Tissue-specific *SLC25A3 *transcripts**. The expression plot shows the mean log 2 intensity signals (with standard error bars) of core probesets targeting *SLC25A3 *exons in the muscle and non-muscle tissue (top figure) and thyroid compared to non-thyroid tissue (bottom figure). The probesets are plotted from left to right by genomic location (5' to 3'). The horizontal dashed line shows the mean log2 intensity of the negative control probesets. Probesets with intensities below this line are most likely unexpressed. In this case these probesets are targeting either intronic regions or UTRs (coloured in orange). Ensembl transcripts for *SLC25A3 *are shown below the plot. Probesets with Benjamini-Hochberg-corrected p-values less than 0.0001 are indicated by black arrows. Exon 3A appears to be retained in muscle and thyroid tissues while exon 3B appears to have lower expression in the muscle.

*SLC25A3 *codes for the mitochondrial phosphate-carrier protein, PiC. Two probesets, 3427827 and 3427830 (ranked 2^nd ^in the muscle vs. non-muscle comparison of the filtered dataset B), mapping to the *SLC25A3 *gene had Benjamini-Hochberg-adjusted p-values less than 0.0001 in the muscle vs. non-muscle comparison and the heart vs. non-heart comparison (Table 2). Probeset 3427827 was also significant in the thyroid vs. non-thyroid analysis. The two probesets map to exon 3A and exon 3B, respectively (Figure 3). Without any prior knowledge about transcript structures, our results would indicate that exon 3A is expressed only in muscle, heart and thyroid tissues and exon B is expressed in all tissues. However, it is known that exon 3A and 3B are two mutually exclusive exons that give rise to Ensembl transcripts ENST00000228318 (contains exon 3A) and ENST00000188376 (contains exon 3B). Exon 3A was found to be highly expressed in heart, muscle and thyroid tissues but had very low expression in all other tissues. All exons had highest expression in heart and muscle tissue, with the exception of exon 3B which had the lowest expression in these two tissues (Figure 3). Taking into account known transcript structures, the expression plots and significant probesets suggest that transcript ENST00000228318 (contains exon 3A but not 3B) is expressed in heart, muscle and thyroid but not in the other tissues, and ENST00000188376 (contains exon 3B but not 3A) is ubiquitously expressed but is not the major transcript in heart and muscle. In thyroid tissues both transcripts may be expressed in roughly equal amounts. Previous studies in mammals have shown that PiC has two distinct isoforms, PiC-A and PiC-B [17], which originate from alternatively spliced transcripts that differ by the mutually exclusive exons 3A and 3B. The two transcripts have displayed different substrate affinities and transport rates *in vitro *[18]. The exon array results are concordant with Mayr *et al*. [19] who showed that PiC-A, the transcript containing exon 3A, has tissue-specific expression in heart and muscle, while PiC-B is expressed ubiquitously.

#### Intersectin 1 (*ITSN1*)

The ITSN protein functions in clathrin-mediated endocytosis and in MAP kinase signalling. Nine probesets mapping to the terminal 11 exons of *ITSN1 *had Benjamini-Hochberg-corrected p-values less than 0.0001 in the cerebellum vs. non-cerebellum analysis of filtered dataset B (Table 2). The expression plot of *ITSN1 *(Additional file 2) clearly showed that the exons at the 3' end of the gene are expressed only in cerebellum. There are two major *ITSN1 *transcripts described in mammals: a ubiquitously-expressed short transcript and a brain-specific long transcript which arises due to brain-specific alternative splicing in a stop codon [20]. The proteins coded by the two transcripts both contain five Src homology 3 (SH3) domains, two Eps15 homology (EH) domains and an apha-helix-forming domain. The longer brain-specific transcript encodes three additional domains (a guanine-nucleotide exchange factors domain, a pleckstrin homology domain and a C2 domain) [20].

#### Kinesin family member 1B (*KIF1*)

**Figure 4:**
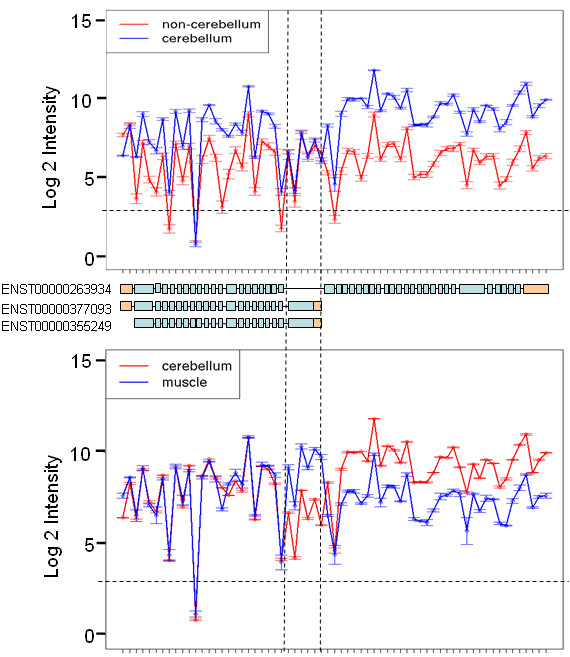
**Tissue-specific *KIF1B *transcripts**. The expression plot shows the mean log 2 intensity signals (with standard error bars) of core probesets targeting *KIF1B *exons in the cerebellum and non-cerebellum tissues (top) and cerebellum compared to muscle tissue (bottom). The horizontal dashed line shows the mean intensity of the negative control probesets. Exons with signal below this are likely to be unexpressed. Most exons have higher probeset signals in the cerebellum except for the 5' UTR (coloured in orange) and the terminal exon of the short transcript (marked by vertical dashed lines) which suggests that this exon has much lower expression in cerebellum. All exons common to the long and short transcripts have the same expression levels in muscle and cerebellum. But exons unique to the long transcript have higher expression in the cerebellum tissue while exons unique to the short transcript have higher expression in muscle.

The KIF1B protein belongs to the kinesin superfamily which are microtubule-dependent molecular motors involved in important intracellular functions such as organelle transport and cell division. Five out of six probesets mapping to an exon (ENSE00001472763) in the *KIF1B *gene had Benjamini-Hochberg-corrected p-values less than 0.0001 in the heart vs. non-heart, cerebellum vs. non-cerebellum (Table 2), muscle vs. non-muscle and thyroid vs. non-thyroid comparisons (data not shown). There are several known isoforms of KIF1B, which are either the long or short forms [21]. The short isoforms lack more than twenty exons from the 3'end and have a terminal 3' exon (ENSE00001472763) that is absent from the long isoforms. ENSE00001472763 was found to be retained in muscle, heart and thyroid but spliced out in cerebellum. Figure 4 shows that all the probesets had a higher intensity signal in the cerebellum tissue compared to non-cerebellum samples, with the exception of the probesets mapping to the 5' UTR and those mapping to ENSE00001472763. Figure 4 shows the exons common to both the long and short isoforms have the same expression levels in cerebellum and muscle. However, exons unique to the short isoforms have higher expression in muscle while exons unique to the long isoform have higher expression in cerebellum. The results indicate that the short transcript is dominant in the heart, muscle and thyroid while the long transcript is dominant in cerebellum. Other tissues may have similar expression for both the long and short transcripts. The KIF1 proteins share a conserved motor domain at their amino-termini. The c-terminal sequences differ widely and are thought to be responsible for their cargo-selection specificity [22,23]. The two isoforms KIF1Balpha (short isoform) and KIF1Bbeta2 (long isoform) differ by the C-terminal cargo-binding domain. The analysis of the exon array data indicates that the dominant transcript in cerebellum tissue has an extended C-terminus (long transcript). Nakumara *et al*. [22] showed that the longer KIF1Bbeta2 transcript was detected in all rat tissues examined, including kidney, liver, spleen, ovary and heart, but was significantly abundant in brain.

#### Inner membrane protein, mitochondrial (*IMMT*)

*IMMT *codes for mitofilin (heart muscle protein) and is thought to play a role in controlling mitochondrial cristae morphology (23). Probeset 2562711 was the most significant splice event in the heart vs. non-heart in the LIMMA analysis of the filtered dataset B (Table 2). It maps to exon 6 of the *IMMT *gene. All the probesets had the highest intensity signal in the heart except for the one targeting exon 6 which had the lowest expression in the heart samples (Additional file 3). There are 3 known Ensembl transcripts for this gene. Transcript 1 (ENST00000254636) and transcript 2 (ENST00000377310) differ only by exon 6 (ENSE00000768006; transcript 2 lacks this exon) [24]. The third transcript (ENST00000398211) contains exon 6 but lacks several other exons. The exon array data suggest that the dominant transcript in heart tissues lacks exon 6, and is therefore likely to be ENST00000377310.

We chose the above genes as the expression pattern could be mapped to know transcript structures. *WNK1*, *SLC25A3 *and *IMMT *were chosen as examples as they contained at least one probeset that ranked within the top 20 most significant probesets in the respective comparisons (when using the filtered dataset A). However, *KIF1B *and especially *ITSN1*, though they had significant p-values, were ranked much lower. This illustrates that it is essential to manually inspect all significant probesets, however large the list, as real splice events will not always be ranked highly by the LIMMA analysis. Both these genes have a much larger number of differentially spliced exons than *WNK1*, *IMMT *and *SLC25A3*. This undoubtedly affects the estimation of accurate gene signal estimates, and in turn the SI, under-estimating the extent of differential splicing. This highlights one of the pitfalls of the SI approach.

A similar study was done on a tissue panel data set (GEO dataset GSE5791) using the Affymetrix Research Exon Arrays [12]. The design of this array differs to that of the HuEx 1.0ST arrays as they consisted of a GeneChip array set of 4 chips and each probeset had up to 4 perfect match (PM) and mismatch (MM) probe pairs. The data set consisted of 6 brain tissues (including cerebellum) and 9 non-brain tissues (including heart, kidney, liver, muscle and testis). Their analysis included all core, extended and full category probesets. The data was normalised using probesets from 71 empirically derived housekeeping genes shown to be consistent across many tissues. To identify significant probesets, multiple t-tests were applied to the gene-normalised exon intensities and an un-corrected p-value cut-off of 0.05 was used to identify significant probesets. Gel profiles of the top-ranking probesets as well as probesets with the highest SI in the brain vs. non-brain comparison were validated by RT-PCR. To check if any of the RT-PCR validated cerebellum-specific splice events were significant in our cerebellum vs. non-cerebellum analysis we had to first map the probeset ids from the Affymetrix Research Exon Array to the corresponding HuEx1.0 ST array probeset ids. We obtained the sequence covered by the probeset (from the 5' most probe to the 3' most probe) from the GEO Platform annotation (GPL4253) and did a BLAT search against the Ensembl database (NCBI Build 36) to obtain the genomic location of the target regions. We then used the X:MAP genome browser to identify HuEx 1.0ST probesets targeting these regions. Our search was restricted to exons targeted by HuEx 1.0ST core probesets as our analysis was done only on core probesets. We were able to map HuEx 1.0ST core probesets for 22 of the cerebellum-specific RT-PCR validated splice events. Though the array platform, tissue panel, pre-processing and analysis methods used by Clark *et al. *[12] were different to the dataset and analysis used in this paper, 13 out of these 22 probesets had Benjamini-Hochberg-corrected p-values less than 0.0001 and 19 had Benjamini-Hochberg-corrected p-values less than 0.05 in our SI/LIMMA analysis of the unfiltered data (Additional File 4).

We also looked at results from a recently published analysis method by Purdom *et al. *[25] called FIRMA (Finding Isoforms using Robust Multichip Analysis). FIRMA scores each exon as to whether its probes systematically deviate from the expected gene expression level. They specifically looked at exons which were previously validated by Das *et al. *[26] as being enriched in muscle and heart tissue as well as reported other top scoring probesets. Out of the 11 core probesets presented by Purdom *et al.*, FIRMA scored highly for 9 in the heart and/or muscle tissue. All 9 of these had Benajmini-corrected p-values less than 0.0001 in our muscle vs. non-muscle and/or heart vs. non-heart SI/LIMMA comparison (Additional file 5).

### Using Pearson's correlation to identify differentially spliced genes

Plotting signal intensities of probesets across a gene provided a better understanding of the nature of the data. We found that for large proportion of genes the probeset signals were quite variable, but the pattern of expression was highly correlated in the different sample groups. We hypothesise that in the absence of splicing the probeset expression pattern between two groups should be highly correlated, with a Pearson correlation coefficient close to 1. Differences in splicing and therefore differences in probeset signal pattern between the two groups will result in a decrease in the gene's correlation.

**Figure 5:**
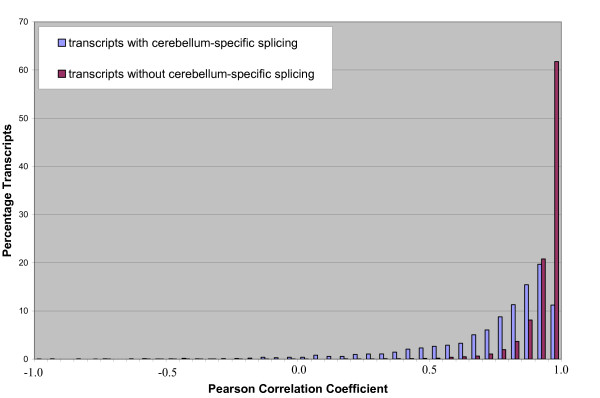
**Pearson Correlation Coefficients**. Transcripts were considered to have cerebellum-specific splicing if they contained at least one probeset with a Benjamini-Hochberg-corrected p-value less than 0.0001 in the cerebellum vs. non-cerebellum SI/LIMMA comparison. Over 80% of transcripts clusters that were considered to have no cerebellum-specific splicing (purple) had correlation coefficients more than 0.9 (more than 60% had coefficients more than 0.95), whereas only 30% of transcript clusters with cerebellum-specific splicing (blue) had correlation coefficients more than 0.9.

To test this hypothesis, for each tissue comparison (i.e. breast vs. non-breast, liver vs. non-liver etc.) we calculated the Pearson correlation coefficient for each transcript cluster (gene) in the filtered dataset A (14180 transcript clusters) using the mean probeset log2 intensities (signals from replicates were averaged). Transcript clusters that contained only one core probeset were removed as no correlation coefficient could be calculated and transcript clusters that contained only two probesets were also excluded, as these had a correlation coefficient of 1 or -1. Over 80% of transcript clusters that showed no cerebellum-specific splicing (did not contain any probesets with Benjamini-Hochberg-corrected p-values less than 0.0001 in the cerebellum vs. non-cerebellum comparison) had a correlation coefficient of 0.90 or higher (94% had a correlation coefficient greater than 0.8), while only around 30% of the transcript clusters that had cerebellum-specific splicing (contained at least one significant probesets in the cerebellum vs. non-cerebellum LIMMA analysis) had a correlation coefficient of 0.90 or higher (Figure 5). Similar distributions were observed for the other tissue comparisons (data not shown). Though the correlation coefficient can be calculated from 3 or more probesets, it is important to note that low correlation coefficients for genes with a large number of exons are more reliable than those for genes with only a small number of exons.

We looked at the correlation coefficients across all 11 comparisons for the 5 genes mentioned above. In all 5 genes the lowest correlation coefficients coincided with the comparisons that contained the most significant probesets (Table 3). For example, *ITSN *is known to have a cerebellum-specific transcript which is much longer than the dominant short isoform expressed in other tissues. All of the tissue comparisons for *ITSN *had a Pearson correlation coefficient above 0.90 except for the cerebellum vs. non-cerebellum comparison, where the correlation coefficient was 0.56. As expected, genes with large number of exons being differentially spliced (*KIF1B *and *ITSN1*) had much lower correlation coefficients than genes with single or few exons being differentially spliced (*IMMT *and *SLC25A3*). With *SLC25A3 *the correlation coefficients in the heart and muscle tissue comparisons are 0.9302 and 0.9412 respectively, while all other tissues had coefficients higher than 0.96. Though this is a small difference it is a significant one. We compared all the correlation coefficients for *SLC25A3 *to its highest correlation coefficient (0.98753 in the liver vs. non-liver comparison) using a statistical test for the significance of the difference between two coefficients using a Fisher r-to-z transformation [27]. The heart and muscle correlation coefficients had z-scores greater than 2 (2.76 in heart and 2.48 in muscle) while all other tissue comparisons had z-scores less than 1.4. The SI/LIMMA correctly identified thyroid-specific splicing of *SLC25A3 *but the correlation coefficient for the thyroid vs. non-thyroid comparison was not significantly different. This may be due to a combination of only a single exon being differentially spliced between thyroid and non-thyroid and the fact that the muscle and heart tissues show the same splicing but are included in the non-thyroid group, giving a mixture of signals for the non-thyroid group. This is also an issue with the SI/LIMMA approach, but can be overcome by carrying out an all-versus-all comparison instead of grouping tissues together and then looking for significant probesets common in all comparisons.

**Table 3:** Correlation Coefficients

	ITSN	IMMT	KIF1B	SLC25A	WNK1
breast vs. non-breast	0.9889	0.9734	0.9473	0.9770	0.9709

cereb. vs. non-cereb.	**0.5605**	0.9823	**0.7727**	0.9661	0.9764

heart vs. non-heart	0.9711	**0.8616**	**0.6756**	**0.9302**	0.9410

kidney vs. non-kidney	0.9656	0.9901	0.9215	0.9799	**0.8768**

liver vs. non-liver	0.9766	0.9452	0.9197	0.9875	0.9793

muscle vs. non-muscle	0.9799	0.9834	**0.8285**	**0.9412**	0.9771

panc. vs. non-panc.	0.9158	0.9772	0.9333	0.9747	0.9427

prost. vs. non-prost	0.9807	0.9940	0.9655	0.9786	0.9431

spleen vs. non-spleen	0.9852	0.9790	0.8928	0.9739	0.9769

testes vs. non-testes	0.9864	0.9960	**0.8198**	0.9717	0.9827

thyroid vs. non-thyroid	0.9889	0.9734	0.9473	0.9770	0.9709

z-score between highest and lowest coefficients	9.71	4.42	6.68	2.76	5.05

Pearson correlation coefficients of the 5 genes (rounded to 4 significant digits) calculated for all eleven tissue comparisons using the unfiltered dataset. Values in bold indicate that the gene had a significant p-value (corrected p-value < 0.0001) in that SI/LIMMA group comparison. The highest and lowest correlation coefficients for each gene were found to be significantly different (all had z-scores greater than 2).

The correlation approach is based on the assumption that the probeset expression varies within a gene but the pattern is maintained in the absence of splicing. However, we have seen a small number of genes where there is little variation in the probeset expression level. These genes tend to have low very correlation coefficients when very slight changes in intensity, likely due to noise, occur. However, these cases are few and can be easily identified and removed by looking at the probeset expression variance for each gene.

## Discussion

The aim of this paper was to propose an analysis workflow for analysing differential splicing using freely available software packages that have already been developed and widely used for gene expression analysis. The LIMMA package provides a framework for analysing experiments with multiple sample groups and provides robust statistics, but as yet has not been applied to exon array data to identify alternative exon splicing analysis. We applied LIMMA to an Affymetrix exon array tissue panel dataset to identify tissue-specific splicing. The main issue with the SI approach is obtaining accurate estimates of gene expression. During this process we explored the data extensively using expression plots and came to the realisation that probeset signals within a gene, though variable maintained the same pattern in all biological groups. Differential exon splicing between two groups introduced differences in the signal patterns of probesets within a gene, with the patterns reducing in similarity when exons were differentially spliced. This led to the concept of using a Pearson correlation coefficient to identify genes with differential splicing between two biological groups, where genes with lower correlation coefficients are more likely to be differentially spliced between two biological groups. This approach avoids the issue of inaccurate gene estimates.

Our initial LIMMA analysis of the data was done on unfiltered data and it became obvious from looking at expression plots that a large number of highly ranked probesets were false positives. It was therefore necessary to introduce filtering steps prior to the LIMMA analysis of the gene-normalised exon intensities which greatly improved the rankings of true positives. The filtering steps taken here are specific for this data set and our objective. We recommend removing low/unexpressed probesets and genes. Unfortunately, there is no direct way of determining whether a gene is expressed or not. We observed that the RMA intensity distribution consisted of two peaks. The first peak generally contains negative controls and noise, while the second peak contains most of the signal. Our lower quartile filtering steps were aimed at reducing the noise, by removing genes falling in the first peak (shown in figure 2). Removing 25% of the data is very stringent, but our objective was to reduce the false positive rate and this will inevitably mean a trade-off with the loss of true positives. The proportion of the data filtered will be dependent on the dataset, as the intensity distributions are likely to vary. We do not recommend only analysing low variant genes. However, analysing this subset separately can help identify unambiguous splicing events.

The significance ranking of probesets was found to be lower when an exon was retained or spliced out in several tissues. For example, in *SLC25A3 *exon 3A is retained in heart, muscle and thyroid but spliced out in the rest of the tissues (Figure 4). The probeset mapping to this exon is ranked much lower than the probeset mapping to exon 3B even though the expression plot shows a much larger change in intensity for exon 3A between muscle and non-muscle (Figure 4). This is because the heart and thyroid tissues were grouped with the rest of the non-muscle tissues, resulting in a higher mean intensity and higher within-group variance for exon 3A in the non-muscle group, leading to a less significant p-value. This is also the case for *KIF1B *where some tissues are expressing the short transcript while others the long isoform. For *KIF1B *this is also evident in the correlation coefficients which range widely between the different comparisons. To overcome this, an all-vs-all comparison may be more appropriate, where one tissue is compared to another tissue individually. Despite these issues, such differential splice events do pass the strict p-value threshold and this indicates that the probesets ranked lower down are as important as the ones ranked highly with the most significant p-values.

The LIMMA approach also appeared to work better when a gene had only a single or very few exons differentially spliced. Genes that had a large number of differentially spliced exons (e.g. *ITSN *and *KIF1B*) were ranked much lower. This is likely due to the effect of splicing on the gene signal estimation. If a gene has equal expression in two samples, its gene signal estimation by the summarisation algorithms in the absence of splicing will be the same for both samples. However, if there are many exons differentially spliced between the two groups, some probesets will have high signals in one group (where the exons are retained) but low expression in the other group (where the exons are spliced out). The algorithms may misestimate the gene signal to be lower in the group where there are many exons spliced which in turn will affect the gene-normalised exon values and may result in less significant p-values. An advantage of the correlation coefficient over methods such as SI is that it does not require gene level estimates, which can be inaccurate, especially when there are a large number of exons differentially spliced within a gene. The correlation coefficients for genes with many differentially spliced exons were much lower and these genes would therefore rank better than genes with single exon splicing. For the 5 example genes, the lower correlation coefficients coincided with the splice events identified by SI/LIMMA. The coefficients were much lower for *ITSN *and *KIF1B*, where multiple exons were differentially spliced.

Though the use of correlation coefficients has not been studied extensively, the preliminary results suggest that it could be used as an informative measure to identify differentially spliced genes. We found that 94% of genes not detected to have differential splicing by the LIMMA analysis had a correlation coefficient greater than 0.8. Therefore, genes with correlation coefficients less than 0.8 are most likely to undergo differential splicing. Correlation coefficients higher than 0.8 could either indicate no splicing or few splicing events. It would be difficult to differentiate between these two using just correlation. The p-values from LIMMA and the gene correlation coefficients therefore should be used together as an indication of whether a gene is likely to be differentially spliced and what the extent of splicing is likely to be.

It is important to emphasize that regardless of the method used to identify differential splicing, the splice events must be manually inspected with the help of expression plots and the use of genome browsers such as X:MAP, where array probesets can be mapped to transcript information. We presented our results as an HTML table with links to all the required information (Additional file 5), making it is easier to inspect the results and separate false positives and ambiguous splice events from obvious tissue-splice events. Analysing such plots highlighted the difficulty in mapping exon expression data to know transcript structures. It is also not always clear whether the expression pattern observed is as a result of novel transcripts or a mixture of transcript structures being expressed simultaneously, unless prior knowledge of exon expression is known. The results suggest that there are a large number of transcript variants yet to be discovered, even when using only core annotated exons.

It may be desirable to analyse the full annotation probesets in order to identify novel splice events and confirm expression of predicted exons. However, including these probesets increases noise as a large number of predicted exons will not be real and will not be expressed in all samples. The false positive rate when using the full annotation is expected to be much higher and stringent filtering becomes crucial when including these probesets. This is evident in intensity distributions for full datasets (not shown), where the peak at low intensities is much larger than in the core dataset. In this case a larger proportion of the data would need to be filtered to remove unexpressed/noisy probesets and genes. As for the correlation coefficient, using extended and full data will increase the number of data points from which to calculate correlation. There are many genes which have only a few core probesets but several extended and full probesets. If all of these are used it may avoid artificially low correlation coefficients. However, the degree of splicing may also be underestimated if the majority of these probesets are not measuring expressed exons. One way of avoiding this is to use the full dataset but to filter the data to remove unexpressed probesets. This way predicted exons that are expressed will still be retained.

There are several exons that are represented by more than one probeset. We have seen several cases from the SI/LIMMA analysis, especially in the terminal exons, where some probesets mapping to the same exon are significant but others are not. These could be as a result of alternative exon splice sites or alternative UTRs. The correlation approach could potentially be used for identifying alternative acceptor and donor sites by calculating a correlation coefficient for each exon rather than a gene. For this application you would need to use the probe-level data. If an exon has a low correlation coefficient this may be indicative of an alternative splice site. However, it is important to bear in mind that analysis with probe-level data is much more computationally intensive and may not be feasible for current standard desktop computers.

### Further Work

One aspect that needs to be explored is the data filtering. The filtering approach used is not very sophisticated and it is likely that viable splice candidates may be filtered out using a quartile filter. The use of the Affymetrix detection above background (DABG) algorithm to filter absent probesets and genes may be a more suitable option which will be explored in the future. Most of our effort will, however, be put into exploring the use of correlation coefficients for identifying differential splicing, particularly looking at the genes in the overlapping region of the correlation coefficient distributions, which may be false positives or false negatives. Another factor to consider when calculating correlation coefficients is the number of exons/probesets in the gene. If a gene has only 7 exons and one exon is differentially spliced the correlation coefficient will be lower than a gene with 14 exons and only a single exon differentially spliced. The number of exons can be taken into account by using z-scores to determine if two correlation coefficients are significantly different or is the difference simply due to the difference in the number of exons. Z-scores can be used in the case where there are multiple biological groups and comparisons and may be a better alternative for selecting genes instead of correlation coefficient cut-off. But in the case of a single comparison between two groups a correlation coefficient threshold would have to be used for selecting differentially spliced genes. In the mean time using a combination of p-value and correlation coefficients would appear to be an efficient way to select genes that are most likely to be differentially spliced.

## Conclusion

We can confidently say our approach using SI/LIMMA can successfully identify differential exon splicing, but lack of any gold standard data set makes it difficult to benchmark this method and any other method without wet-lab validation. However, we were able to use previous experimental studies to support our results. It is also difficult to directly compare the correlation results as it gives a measure for each transcript cluster (gene) while all other methods, including SI/LIMMA, give p-values for a probeset (exon). But we have shown that the distribution of correlation coefficients in transcripts without splicing is significantly different to the distribution in transcripts with splicing as identified by the SI/LIMMA analysis. Thus low correlations are indicative of differentially spliced genes. Though further work would be necessary to develop the use of correlation coefficients into a complete analysis approach, the preliminary results demonstrate their usefulness for identifying differentially spliced genes.

## Availability and requirements

The R scripts are available upon request from the authors.

## Methods

### Dataset

The dataset consists of 33 arrays: 11 human tissues (breast, cerebellum, heart, kidney, liver, muscle, pancreas, prostate, spleen, testes and thyroid) with three assay replicates per tissue. Samples are from a commercial source. The dataset is available for download from the Affymetrix website: 

### Data Summarisation and Normalisation

The exon-level and gene-level data was generated from the CEL files using the Affymetrix Power Tools (APT). The library files used were: HuEx-1_0-st.v2.r2.dt1.hg18.core.ps, HuEx-1_0-st.v2.r2.dt1.hg18.core.mps, HuEx-1_0-st-v2.r2.pgf, HuEx-1_0-st-v2.r2.clf and HuEx-1_0-st-v2.r2.antigenomic.bgp. RMA background correction, quantile normalisation and RMA summarisation was used to generate the exon and gene-level data.

### Filtering Steps

In the Affymetrix transcript annotation file HuEx-1_0-st-v2.na24.hg18.probeset.csv the 'crosshyb_type' column labels probesets as either 1 (unique – perfectly match only the target sequence), 2 (similar – perfectly match more than one sequence) or 3 (mixed – perfectly or partially match more than one sequence). Probesets classified as 2 (similar) or 3 (mixed) were filtered out in the exon-level data.

After filtering cross-hybridising probesets, low expression probesets were filtered. The probesets in each sample were ranked by log intensity (lowest expression is ranked 1). The rank product for each probeset was then calculated using the following formula:

$$RP(g)={\left(\Pi_{i=1}^{k}r_{g,i}\right)}^{1/k}$$

where *k *is the number of samples and *r*_*g*, *i *_the rank of probeset *g *in the *i*^*th *^sample.

The probesets in the quartile with the lowest rank products were removed. The same method was applied to the gene level data to filter out transcript clusters with the lowest expression across all samples. In this case *g *would be the transcript cluster. This dataset is known as **filtered dataset A**.

A subset of the filtered dataset A was generated by selecting the transcript clusters with the least sample-to-sample variation. The transcript clusters that fell in the quartile with the highest variation were excluded from this subset. This is known as **filtered dataset B**. This effectively removed genes with large expression differences between groups as these had a tendency to produce false positives.

### Alternative Splicing Analysis

Probesets were mapped to their corresponding transcript clusters using the HuEx-1_0-st-v2.r2.na24.hg18.probeset.csv annotation file. For each probeset in each sample, the 'gene-normalised exon values' were calculated, which are simply the log2 probeset over transcript cluster intensity ratios:

$$GNE(p)=log_{2}\left(\frac{e_{p}}{g_{p}}\right)$$

where GNE is the gene-normalised exon value for probeset *p*, e_*p *_is the exon-level summary for *p *and *g*_*p *_is the gene-level summary for the transcript cluster (gene) to which *p *maps. Any probesets ids with no corresponding transcript cluster ids or vice versa will be removed from further analysis.

LIMMA was applied to the gene-normalised exon values from the unfiltered, filtered datasets A and B. Eleven comparisons were made simultaneously (breast vs. non-breast, heart vs. non-heart, kidney vs. non-kidney etc.) in order to identify tissue-specific exon splicing. The Benjamini-Hochberg method was used to correct the raw p-values for multiple-testing. Probesets with corrected p-values less than 0.0001 were considered significant. The R script is available from .

### Pearson Correlation Coefficient

For each group in each comparison the Pearson correlation coefficient for every transcript cluster was calculated in R using the mean probeset intensities for each group. The R code for the correlation coefficient analysis is available at: .

To test the significance of the difference between two correlation coefficients we used the web based tool at .

### Visualisation & Annotation

Annotation of significant probesets was obtained using the Affymetrix annotation files (HuEx-1_0-st-v2.na24.hg18.probeset.csv) available from the Affymetrix website. HTML results files with links to expression plots, X:MAP, Netaffx and NCBI were created in R.

## Abbreviations

APT: Affymetrix Power Tools; FIRMA: Finding Isoforms using Robust Multichip, Average; LIMMA: Linear Models for Microarray Analysis; PLIER: Probe, Logarithmic Intensity Error; RMA: Robust Multi Average

## Authors' contributions

SS developed the workflow, conceived the idea of the correlation coefficient and performed the data analysis. JP supervised the work. All authors read and approved the final manuscript.

## Supplementary Material

Additional file 1 **Differential Splicing of *WNK1*.** The expression plot shows the mean log 2 intensity signals (with standard error bars) of core probesets targeting *WNK1 *exons in the kidney and non-kidney tissues. The horizontal dashed line shows the mean intensity of the negative control probesets. Exons with signal below this are likely to be unexpressed. All 7 probesets targeting the first 4 exons (marked by horizontal dashed lines) had Benjamini-Hochberg-corrected p-values less than 0.0001 in the kidney vs. non-kidney comparison. Click here for file

Additional file 2 **Differential Splicing of *ITSN*.** The expression plot shows the mean log 2 intensity signals (with standard error bars) of core probesets targeting *ITSN *exons in the cerebellum and compared to non-cerebellum tissues. The horizontal dashed line shows the mean intensity of the negative control probesets. Exons with signal below this are likely to be unexpressed. Nine probesets targeting the last 11 exons (marked by horizontal dashed lines) had Benjamini-Hochberg-corrected p-values less than 0.0001 in the cerebellum vs. non-cerebellum comparison. Click here for file

Additional file 3 **Differential Splicing of *IMMT*.** The expression plot shows the mean log 2 intensity signals (with standard error bars) of core probesets targeting *IMMT *exons in heart and non-heart tissues. The horizontal dashed line shows the mean intensity of the negative control probesets. Exons with signal below this are likely to be unexpressed. Probeset 2562711 was the most significant splice event in the heart vs. non-heart in the LIMMA analysis of the filtered dataset B. It maps to exon 6 of the *IMMT *gene. All probesets have higher signals in heart tissue except for the significant probeset (marked by black arrow). Click here for file

Additional file 4 **P-values and correlation coefficients for RT-PCR validated probesets.** Excel file containing information on the 22 mapped probesets from the RT-PCR validation by Clark *et al. *and the mapped probesets presented by Purdom *et al. *The table displays the raw and Benjamini-Hochberg-corrected p-values from the LIMMA analysis. Click here for file

Additional file 5 **Display of Results. Results were presented as HTML tables containing probesets sorted by significance.** For each probesest, the target gene and its annotation were provided. HTML links were also provided to view the expression plot of the genes, the Netaffx entry for the probeset and transcript cluster ids, the X:MAP genome browser showing the location of the probeset and NCBI gene information. Click here for file
