# Supplementary figures and images for: Identifying differential exon splicing using linear models and correlation coefficients
Source: BMC Bioinformatics. 2009 Jan 20;10:26. doi: 10.1186/1471-2105-10-26 (PMC2636774; doi:10.1186/1471-2105-10-26)

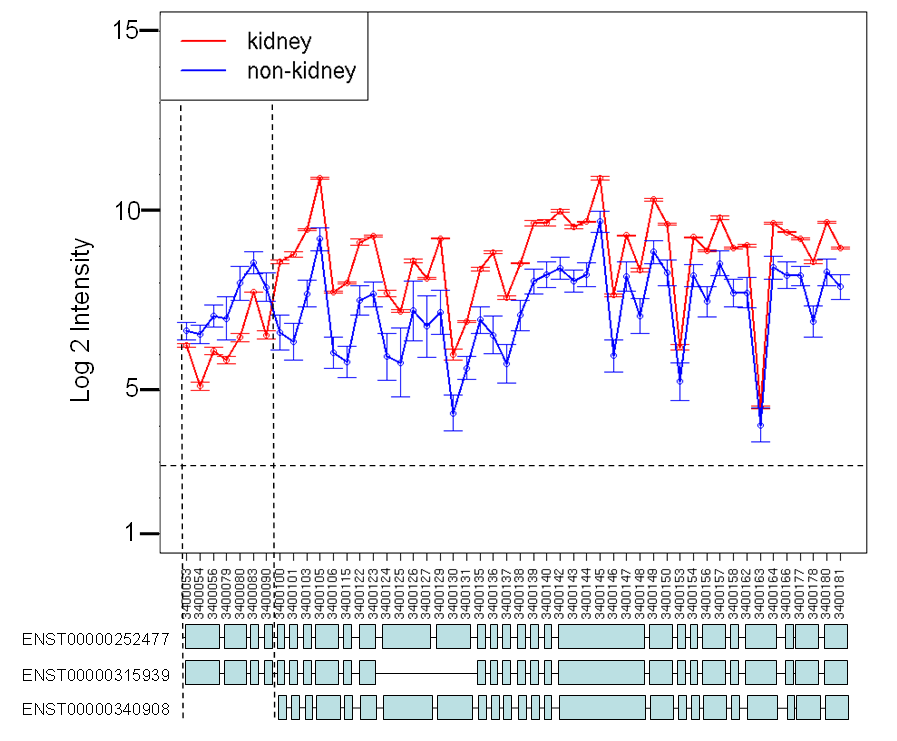

Supplement: Additional file 1 — Differential Splicing of WNK1. The expression plot shows the mean log 2 intensity signals (with standard error bars) of core probesets targeting WNK1 exons in the kidney and non-kidney tissues. The horizontal dashed line shows the mean intensity of the negative control probesets. Exons with signal below this are likely to be unexpressed. All 7 probesets targeting the first 4 exons (marked by horizontal dashed lines) had Benjamini-Hochberg-corrected p-values less than 0.0001 in the kidney vs. non-kidney comparison. [file 1471-2105-10-26-S1.png]

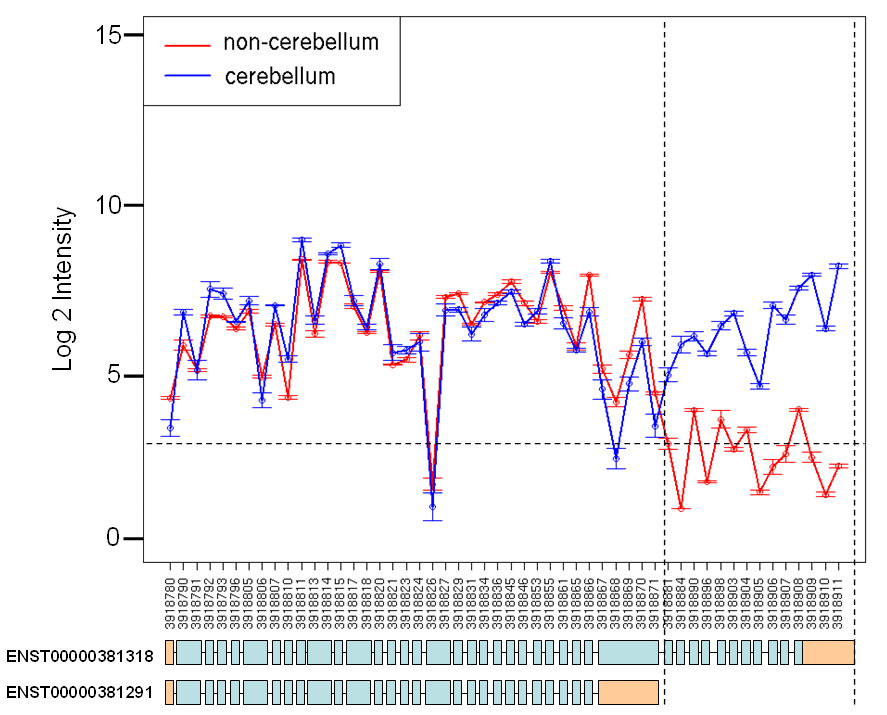

Supplement: Additional file 2 — Differential Splicing of ITSN. The expression plot shows the mean log 2 intensity signals (with standard error bars) of core probesets targeting ITSN exons in the cerebellum and compared to non-cerebellum tissues. The horizontal dashed line shows the mean intensity of the negative control probesets. Exons with signal below this are likely to be unexpressed. Nine probesets targeting the last 11 exons (marked by horizontal dashed lines) had Benjamini-Hochberg-corrected p-values less than 0.0001 in the cerebellum vs. non-cerebellum comparison. [file 1471-2105-10-26-S2.png]

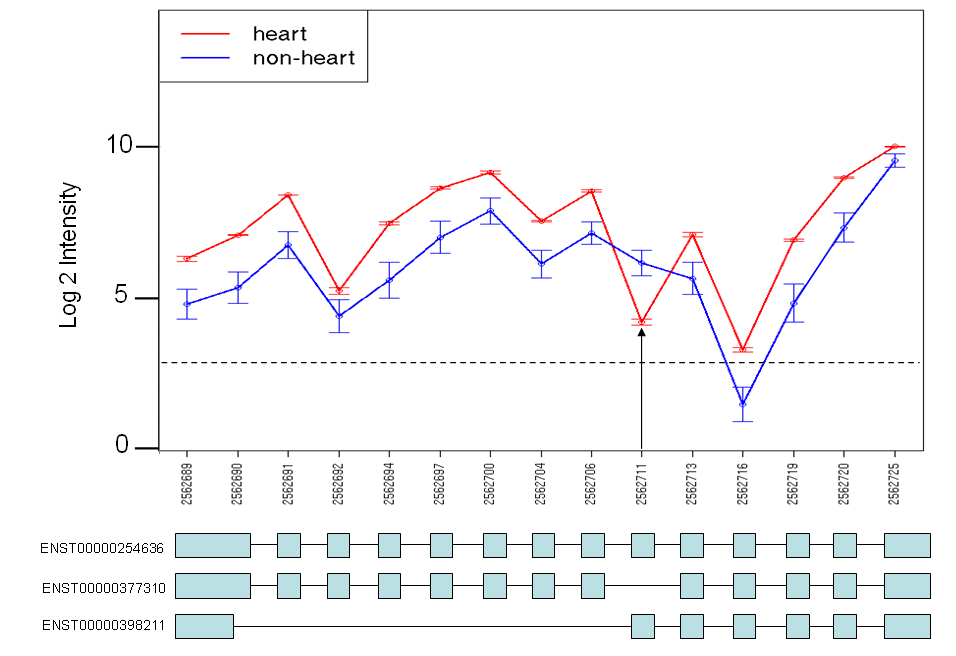

Supplement: Additional file 3 — Differential Splicing of IMMT. The expression plot shows the mean log 2 intensity signals (with standard error bars) of core probesets targeting IMMT exons in heart and non-heart tissues. The horizontal dashed line shows the mean intensity of the negative control probesets. Exons with signal below this are likely to be unexpressed. Probeset 2562711 was the most significant splice event in the heart vs. non-heart in the LIMMA analysis of the filtered dataset B. It maps to exon 6 of the IMMT gene. All probesets have higher signals in heart tissue except for the significant probeset (marked by black arrow). [file 1471-2105-10-26-S3.png]

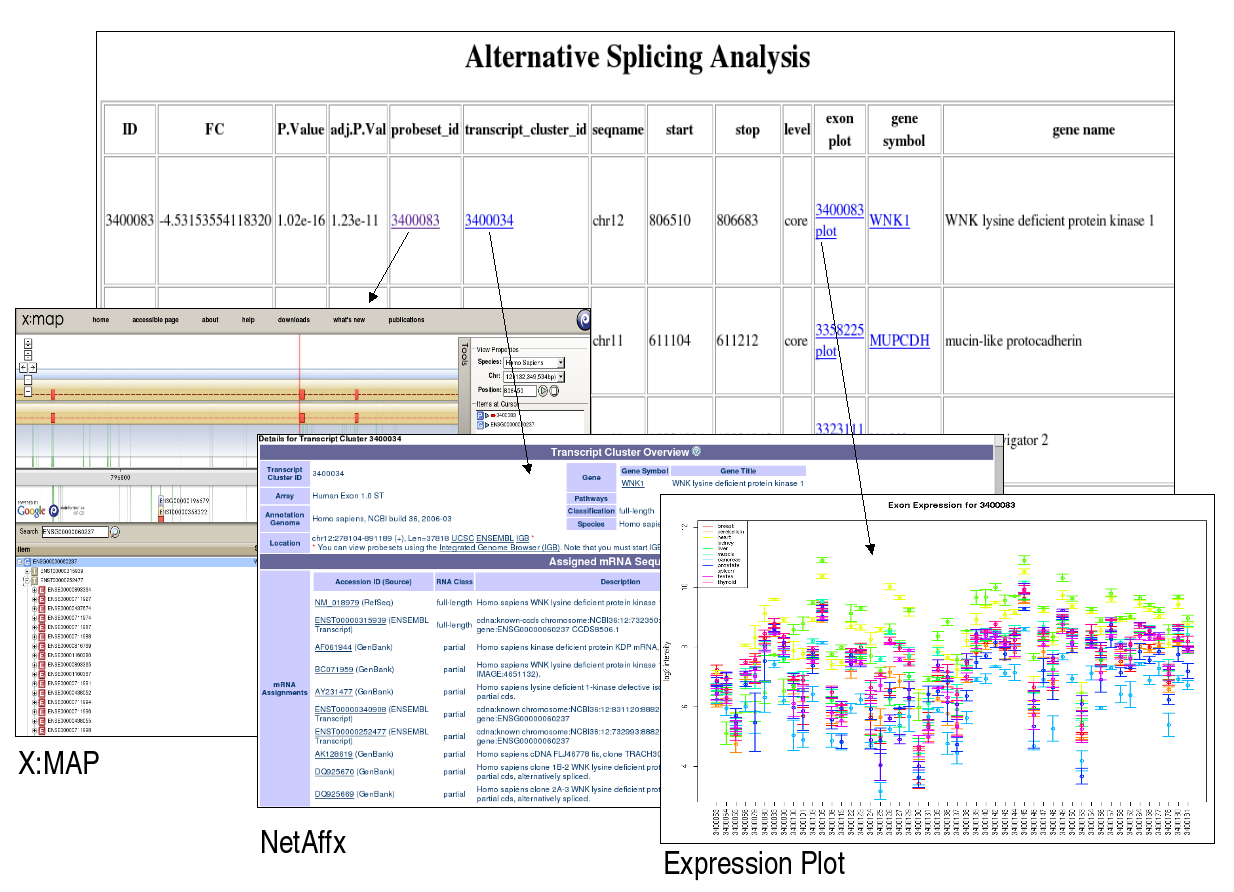

Supplement: Additional file 5 — Display of Results. Results were presented as HTML tables containing probesets sorted by significance. For each probesest, the target gene and its annotation were provided. HTML links were also provided to view the expression plot of the genes, the Netaffx entry for the probeset and transcript cluster ids, the X:MAP genome browser showing the location of the probeset and NCBI gene information. [file 1471-2105-10-26-S5.png]
